# Supplementary figures and images for: Prostaglandin D2 receptor 2 downstream signaling and modulation of type 2 innate lymphoid cells from patients with asthma
Source: PLoS One. 2024 Jul 25;19(7):e0307750. doi: 10.1371/journal.pone.0307750 (PMC11271944; doi:10.1371/journal.pone.0307750)

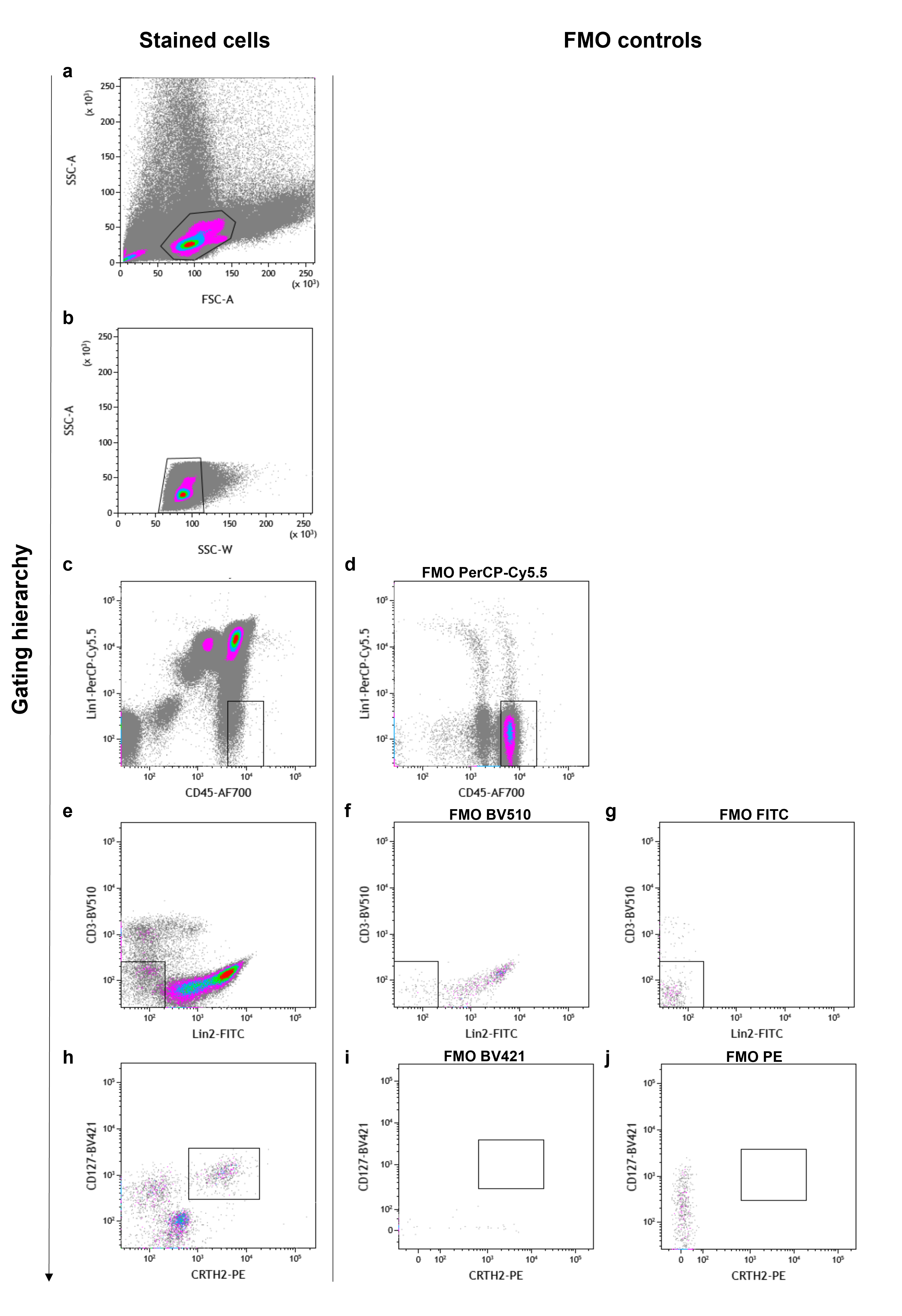

Supplement: S1 Fig — After depletion of CD3-, CD14- and CD19-positive cells from PBMCs via magnetic activated cell separation (MACS), cells were stained with a PerCP-Cy5.5-labeled lineage cocktail 1 (CD4, CD8, CD14, CD16, CD19, CD34, CD123, FcεRI), a FITC-labeled lineage cocktail 2 (CD11b, CD56), CD3-BV510, CD127-BV421, CRTH2-PE and CD45-Alexa Fluor 700. (a) Lymphoid cells were gated by cell size (FSC-A) and cell granularity (SSC-A), and (b) discriminated from doublets (clotted cells) by determination of the time of flight that each cells needed to pass the filter (SSC-W). For further gating, (d, f, g, i, j) gates were set according to FMO controls. (c) CD45high, lineage1-, (e) lineage2-, CD3-, (h) CD127+ and CRTH2+ cells were defined as ILC2 and sorted into 96 U buttom well plates containing 100 μL supplemented medium. Gate boundaries were not altered between participants. (TIF) [file pone.0307750.s001.tif]

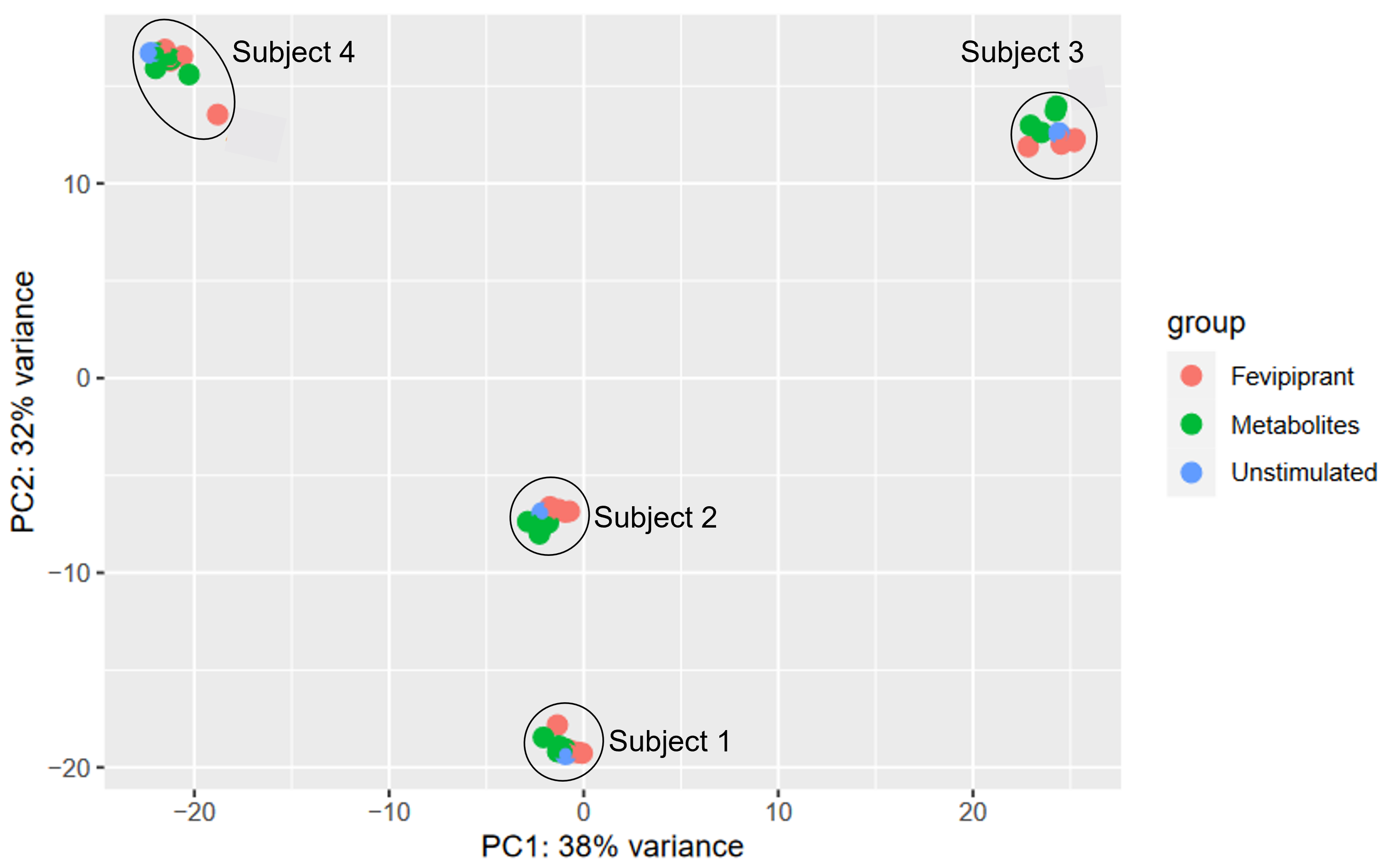

Supplement: S2 Fig — (TIF) [file pone.0307750.s002.tif]

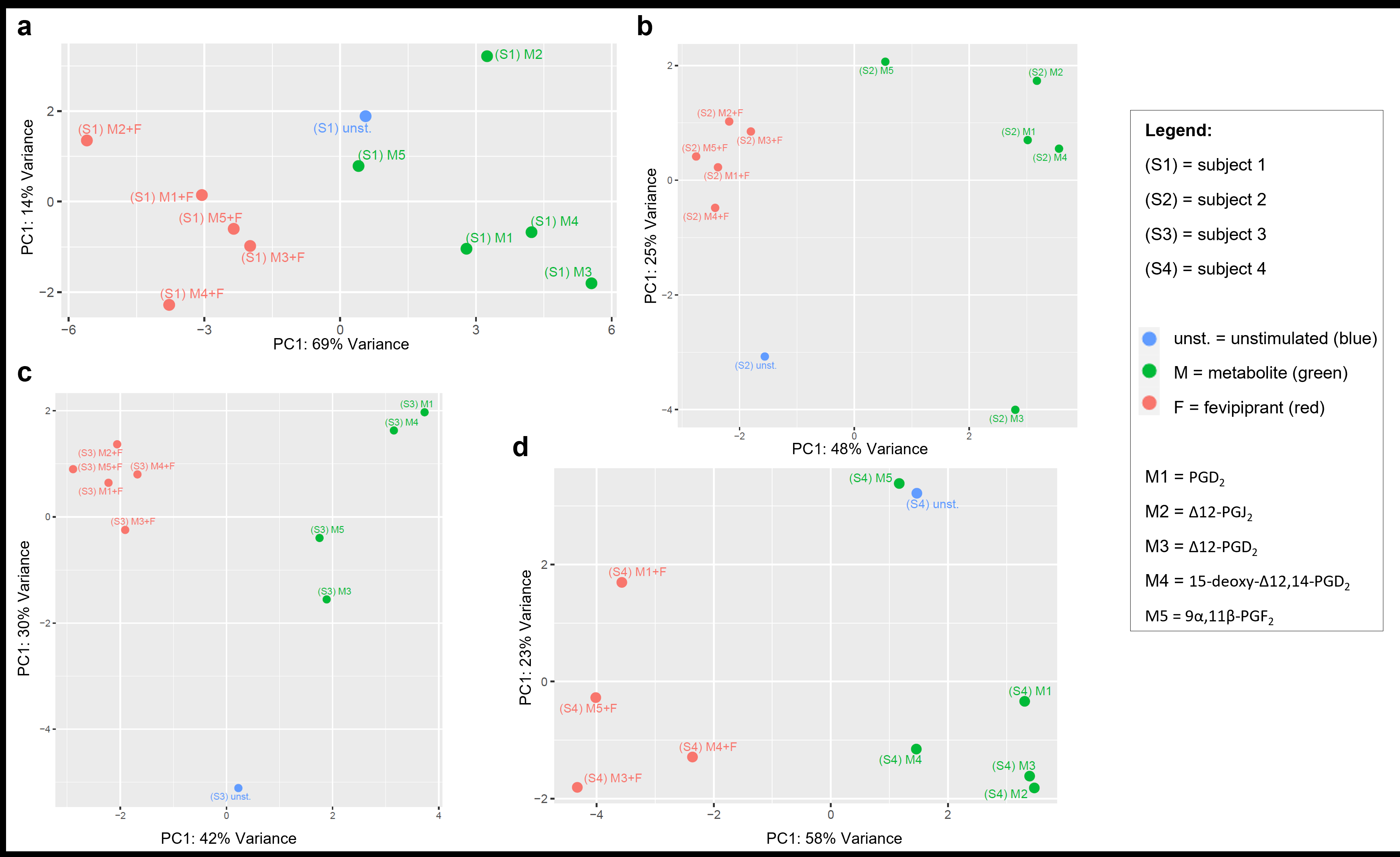

Supplement: S3 Fig — (TIF) [file pone.0307750.s003.tif]
